# Cost-effectiveness analysis of a cluster-randomized, culturally tailored, community health worker home-visiting diabetes intervention versus standard care in American Samoa

**DOI:** 10.1186/s12960-019-0356-6

**Published:** 2019-03-05

**Authors:** Shuo J. Huang, Omar Galárraga, Kelley A. Smith, Saipale Fuimaono, Stephen T. McGarvey

**Affiliations:** 1 Department of Epidemiology, International Health Institute, Brown University School of Public Health, Box G-S -121-2, Providence, RI 02912 United States of America; 2 Department of Health Services Administration, University of Maryland School of Public Health, College Park, MD United States of America; 3 Department of Health Services, Policy, and Practice, International Health Institute, Brown University School of Public Health, Providence, RI United States of America; 4 Tafuna Family Health Centers, Pago Pago, American Samoa

**Keywords:** Community health workers, Cost-effectiveness analysis, Cost-utility analysis, Type 2 diabetes, Behavioral intervention, Direct health care costs, Disease management

## Abstract

**Background:**

Type 2 diabetes mellitus (T2DM) is highly prevalent in American Samoa. Community health worker (CHW) interventions may improve T2DM care and be cost-effective. Current cost-effectiveness analyses (CEA) of CHW interventions have either overlooked important cost considerations or not been based on randomized clinical trials (RCTs). The Diabetes Care in American Samoa (DCAS) intervention which occurred in 2009–2010 was a cluster-randomized, culturally tailored, home-visiting CHW intervention and improved HbA1c levels.

**Objective:**

To analyze the cost-effectiveness of the DCAS intervention against standard care using a RCT in a low-resource setting.

**Methods:**

We collected clinical, utilization, and cost data over 2 years and modeled quality-adjusted life years (QALYs) gained based on the RCT glycated hemoglobin (HbA1c) improvements. We calculated an incremental cost-effectiveness ratio (ICER) from the societal perspective over a 2-year time horizon and reported all costs in 2012 USD ($).

**Results:**

Two hundred sixty-eight American Samoans diagnosed with T2DM were cluster randomized into the CHW (*n* = 104) or standard care control (*n* = 164) arms. The CHW arm had a mean reduction of 0.53% in HbA1c, an increase of $594 in cost, and an increase of 0.05 QALYs. The ICER for the CHW arm compared to the control arm was $1121 per percentage point HbA1c reduced and $13 191 per QALY gained.

**Conclusions:**

Compared to a variety of willingness-to-pay thresholds from $39 000 to $154 353 per QALY gained, this ICER shows that the CHW intervention is highly cost-effective. Future studies of the cost-effectiveness of CHW T2DM interventions in similar settings should model lifetime costs and QALYs gained to better assess long-term cost-effectiveness.

**Trial registration:**

ClinicalTrials.gov, ID NCT00850824. Registered 9 February 2009,

https://clinicaltrials.gov/ct2/show/NCT00850824.

**Electronic supplementary material:**

The online version of this article (10.1186/s12960-019-0356-6) contains supplementary material, which is available to authorized users.

## Introduction

American Samoa is a US territory in the South Pacific with a high prevalence of cardiometabolic non-communicable diseases (NCDs). The prevalence of NCDs, including type 2 diabetes mellitus (T2DM), is disproportionately high in many Pacific Island countries and territories, although American Samoa has the world’s highest national prevalence [1–3]. More than 30% of adults in a total population of approximately 56 000 have T2DM [3], a prevalence more than three times that of the United States of America [4]. Unmanaged T2DM is a major risk factor for cardiovascular diseases, blindness, chronic kidney disease, and mortality, all of which are costly for health systems and societies [5]. Most current T2DM cases are managed using medication alongside behavioral and lifestyle modifications, but the continuity of care from awareness, diagnosis, and adherence to control is often challenging due to numerous cultural and social factors, particularly in low-resource settings [6].

One strategy to promote better chronic disease management is to employ community health workers (CHWs). CHW programs, particularly those that are culturally appropriate, improve clinical outcomes in patients with chronic diseases, including T2DM [7]. A systematic review of CHW interventions focused on improving glycemic control found modest reductions in glycated hemoglobin (HbA1c) levels compared to usual care [8]. CHW interventions may be particularly beneficial for improving T2DM outcomes in medically underserved populations. Low-income African Americans in Baltimore who participated in Project Sugar 2, a culturally tailored behavioral and lifestyle modification intervention, lowered their HbA1c [9]. Minority participants in a CHW study in Michigan also decreased their HBA1c [10], and among a population of low-income Mexican-American adults receiving CHW care, 80% of the cohort that had baseline HbA1c above 9% reduced this measure during the intervention [11].

Furthermore, CHWs improve health utilization, potentially improving the quality of care while lowering costs. The participants in Project Sugar 2 reduced emergency department (ED) visits [12]. In our Diabetes Care in American Samoa (DCAS) study [13], participants who were high ED users at baseline reduced those visits during the intervention year; additionally, participants in the CHW arm of the study increased their primary care utilization during that period [14].

CHW interventions may be more cost-effective than standard interventions, as they generally have lower staffing costs than those using nurses and physicians. This task shifting is critical for low-resource communities. Additionally, previous studies suggest that CHW interventions may result in fewer hospitalizations and increased use of primary care, both of which are associated with decreased medical costs [15]. Despite evidence of both improved clinical outcomes and cost-effectiveness, few studies have conducted cost-effectiveness analyses (CEA) of CHW interventions in low-resource settings. In a 2016 review of 67 chronic disease studies, only 8 studies estimated cost-effectiveness [7], and in a 2010 review of 53 CHW studies, only 6 collected cost data [16].

Of the CHW studies on T2DM that conducted CEAs, few were designed as randomized control trials (RCTs). Two studies showing cost savings lacked control groups. A 2004 study of a T2DM self-management program calculated a cost of $185 per 1% unit reduction in HbA1c, concluding that HbA1c improvement was obtained at a modest cost [17]. Without a comparator, however, we do not know the additional costs or effects, nor can we construct an incremental cost-effectiveness ratio (ICER). Moreover, the study only collected the direct cost of the intervention and did not account for potential changes in resource utilization. The CHW T2DM intervention for Mexican-Americans reported an ICER range of $10 995 to $33 319 per quality-adjusted life years (QALYs) gained across a lifetime [11]. While this is considered highly cost-effective, the study did not include a control group and instead modeled additional costs and effects based upon a usual care population found in a registry.

RCTs that include CEAs have not always collected cost comparators. A CHW intervention in a primarily low-income, African-American population in Baltimore found an ICER of $149 per one percentage point reduction in HbA1c over 1 year compared to physician care [18]. However, that study did not include the opportunity costs of space and equipment used by the CHW arm of the intervention, the costs of physician consultations, or the costs of continued primary, emergency, or hospital care. Therefore, our understanding of the cost-effectiveness of CHW T2DM interventions in low-resource settings is limited by the dearth of RCTs that collect full-cost information. Cost-effectiveness studies need to examine the impact on costs and effects for all material changes related to the intervention beyond direct program costs, including increased or decreased health utilization, forgone wages and work, and lost opportunity costs arising from conducting the intervention [19–21].

Since follow-up periods for most trials and RCTs of T2DM are relatively short, cost-effectiveness is often assessed through validated modeling of costs and QALYs gained over a longer time horizon. Since many cost-effectiveness studies are validated on the same underlying model, there is a risk that cost-effectiveness is over or understated [22–26]. Some of the most cited studies were validated on the United Kingdom Prospective Diabetes Study (UKPDS), which had an initial follow-up period of 10 years [27], plus an additional 10 years of follow-up [28]. The additional follow-up phase for UKPDS found that while HbA1c differences disappeared with the end of the trial protocol, benefits to reduced mortality and CVD risk persisted 10 years after the end of the intervention, suggesting that even temporary glycemic control has long-term benefits for patients [28]. Other frequently cited studies included those with follow-ups ranging from 3.7–5.6 years [29, 30]. In the presence of uncertainty, short-term cost-effectiveness studies based on more directly observed costs and clinical data can help inform decision-makers of the relative value of new interventions.

This study evaluates the cost-effectiveness of the DCAS intervention against standard care using a RCT in the low-resource setting of American Samoa. We hypothesize that a CHW intervention will be cost-effective relative to the standard care in the Samoan study population using multiple willingness-to-pay thresholds.

## Methods

Our study sample consisted of adults over 18, self-identifying as ethnically Samoan, resident in American Samoa and diagnosed with T2DM [13]. Eighty-nine percent of the population is of Samoan ethnicity, and 98% lives on the main island of Tutuila. Per capita gross domestic product (GDP) in 2013 was estimated at $13 000, and minimum wage ranges from $4.58 to $5.99 per hour depending on industry; the main private sector industry is tuna canning, where the minimum wage has been $5.16 per hour since 2015 [31, 32]. Cardiometabolic conditions and risk factors are very high, especially T2DM, as described above. Based on the WHO STEPS survey in 2004, 75% of adults are obese (body mass index > 30 kg/m^2^), 34% have hypertension, 23% have elevated total cholesterol, and 30% smoke cigarettes daily [33].

The public Lyndon Baines Johnson Tropical Medical Center (LBJTMC) provides all hospital services, which include two primary care clinics; ambulatory ophthalmology; ear, nose, and throat (ENT); surgical services; 24-h ED; an intensive care unit (ICU); an acute medicine ward; and a surgery ward. Few American Samoans have health insurance, and the US Centers for Medicare & Medicaid Services (CMS) reimburses LBJTMC with a block grant [34]. All co-pays for services for American Samoans are on a fee schedule.

The DCAS study was based at Tafuna Family Health Center (TFHC), a Federally Qualified Health Center with an independent governing board, which provided space for the research. TFHC receives administrative support from the American Samoa Department of Health (AS DOH).

The DCAS study has been fully described elsewhere [13, 35, 36]. From February 2009 to May 2010, 268 participants with a T2DM diagnosis were cluster randomized into the CHW arm [*n* = 104] or the wait-list standard care arm [*n* = 164], which received the intervention at the end of their 1-year follow-up. We designed culturally relevant and culturally tailored CHW home visit protocols to address behaviors important in managing T2DM. The concepts and methods of adapting a successful T2DM intervention for the Samoan context, as well as details on the content of the adaptation, are described elsewhere [35, 36]. We measured HbA1c at enrollment and at a 1-year follow-up assessment for both arms and collected data on potential mediators including health system use, medication adherence, healthy eating, and physical activity [13, 36]. We estimated the sample size to detect a clinically significant reduction of 0.5% in HbA1c in the CHW arm. Ninety-one percent of participants were retained.

Healthcare utilization was measured through retrospective medical record abstraction of primary care and hospital ED utilization for each participant [14]. The abstraction examined a 2-year period for each participant by comparing the 1 year before enrollment to the 1 year between enrollment and the end of the follow-up. Thus, we implemented a difference in differences (DiD) approach from a baseline period (− 12 to 0 months) for each patient relative to control patients across the intervention time period (0 to 12 months). Data included medication refills, dates and settings of clinic encounters, ED encounters, hospitalizations, lengths of stay (LOS), procedures, and discharge summaries for hospitalizations, comprehensive procedural codes, and medications ordered.

We performed a CEA by calculating the incremental cost-effectiveness of the CHW intervention against standard diabetes care. We limited our timeframe to the 2-year period for which we have actual utilization data from both study arms. Our measure of effectiveness is the difference in change of QALYs between CHW and standard care arms, which allows us to compare DCAS’s cost-effectiveness to a wide range of other interventions. All analysis was done from an intent-to-treat perspective. We evaluated our ICER against multiple willingness to pay thresholds, since—although common and persistent—the $50 000 per QALY gained willingness to pay threshold lacks a strong theoretical grounding [37]. We included the commonly used threshold of $50 000 per QALY gained, two times and three times US GDP per capita per QALY [38], which are $102 902 and $154 353/QALY gained respectively, three times GDP per capita per QALY in the local setting which is $39 000, and study-derived thresholds found in the literature of $66 351 and $111 134 per QALY [38, 39].

We conducted analysis from the societal perspective, which evaluates resource costs to all sectors of society including the health system, payers, and consumers [19, 20]. Our cost measure is the cost of the incremental difference in change in health resource utilization between the CHW and standard care arms. Our broad categories of cost include intervention costs, clinic and hospital ambulatory costs, hospital ED costs, hospital inpatient costs, hospital procedure costs, and patient indirect costs.

For our base case, our measure of effectiveness used the point estimate of HbA1c reduction multiplied by the point estimate of the effect of HbA1c on QALYs gained. Our measure of costs was the point estimates of utilization changes multiplied by cost estimates from financial and administrative reports. We describe data collection and selection below.

We collected selected costs for intervention resources and utilization during the study. In 2013, 2 years after the end of intervention activities, we retrospectively collected exhaustive costs of the healthcare resources that participants used during the 2-year utilization study period, taking advantage of our full access to financial and administrative data as well as utilization reports via electronic medical records. We evaluated costs through activity-based micro-costing, assessing costs per patient visit or per inpatient day by clinic or ward, rather than at an overall level. Overhead costs were step-allocated to activity centers by relevant work management units for each overhead cost center. Work management units included a number of staff for administrative overhead, floor space for plant upkeep and laundry, number of patients for patient service management, and inpatient-equivalent days for clinic services such as the lab. We divided the total cost generated by each ambulatory clinic and the ED by the total number of patient visits applicable to each clinic. This cost per patient visit was then multiplied by the number of visits per patient. We then divided the total cost generated by each hospital ward by the total number of inpatient days applicable to each ward. All costs were converted to 2012 United States dollars (USD).

Variable costs collected during the main phase were estimated from intervention consumables used over 3-month intervals. These costs were then divided by the number of participant visits in that time period to provide a variable cost per visit. Startup capital costs were depreciated over a 5-year useful life. Relevant proportions of startup costs, staff salaries, donated space, and other overhead costs were divided by the number of visits in the busiest month. We selected the busiest month to show overhead costs per visit when the intervention was operating at full capacity, which would be more typical of an ongoing program conducted by the health center. Since TFHC donated space during the intervention research, the office manager provided the opportunity cost of renting the space for a similar use and duration.

TFHC provided annual Health Resources and Service Administration (HRSA) clinic reports including a financial report and comprehensive utilization data. The AS DOH also provided detailed staffing costs.

Care activities at LBJTMC were costed in detail. For fiscal year 2012, we received an audited financial report, floor space by departments, partial reports of capital costs, detailed expenditures for each hospital department, and the number of patients who visited each medical department and LOS for each ward. We received an anonymized staff list by department. Financial information collected for years 2008–2010 served as baseline and the 2011–2012 period served as intervention period data. We estimated the cost of care using the average spending by a medical clinic, ward, or procedural facility for a patient visit (e.g., for an ambulatory clinic, ED, inpatient day, or procedure). We excluded utilization changes in the maternity ward and Ob-Gyn clinic since those charges would be unrelated to changes in HbA1c in our older adult study sample. Direct medical cost details are described in Additional file 1: Table S1.

We estimated patient costs based on the indirect costs of time spent in the intervention or in using medical care. We counted hospitalizations as full days, and outpatient visits at 30 min based upon staff reports. We excluded patient co-pays for medical services to avoid double-counting the economic cost of medical care that should already be captured in expenses for producing medical care. We valued time spent at the 2009 minimum wage of the largest employment sector in American Samoa plus time and a half for overtime.

We estimated QALYs gained or lost in the 2-year timeframe by modeling QALY changes on measured changes in HbA1c levels. We base our QALY estimates on the results of cross-sectional associations between utility weights from surveyed EQ-5D questionnaire states (with US utility preferences applied) and HbA1c levels collected concurrently in a sample of 303 T2DM outpatients age 18 and older from Thailand [34]. The extrapolation of the utility weight is conservative because HbA1c in that study ranged from 4.0 to 15.8%, with a mean of 7.7%, which is lower than our baseline mean of 9.8% [13]. Each additional percentage point of HbA1c was associated with a 0.17 decrease in utility weight. We estimate QALYs gained for the HbA1c reduction over the follow-up year of the intervention from the change in HbA1c. Since we do not have midpoint measures of HbA1c, to calculate QALYs gained, we assumed a linear change from the HbA1c measurement at the time of randomization to the final HbA1c measurement at the 1-year follow-up. We multiplied this change in utility weight by the change in HbA1c and integrated over 1 year. Since we employed a wait-list control design in the RCT intervention study, we cannot analyze the impact of the intervention on HbA1c data beyond our study period. We opted not to apply a model of QALYs gained over a longer time horizon to avoid making assumptions over and above what was actually observed in the RCT.

We conducted one-way sensitivity analyses focused on two assumptions: the reduction of HbA1c and the effect of HbA1c on QALYs gained. HbA1c change was varied within the confidence bounds found in the DCAS main study [13]. QALYs gained were varied by using EQ-5D utility weights for each health state from Japan [40] and by substituting more conservative values for the relationship between HbA1c and QALYs from a cross-sectional type 1 diabetes study that controlled for possible mediators of HbA1c’s effect on QALYs such as medication use and diabetic complications [41].

## Results

A sample of 268 patients (104 in CHW and 164 in usual care) was enrolled in the DCAS. Effectiveness results of the DCAS study are described elsewhere [13]. Table 1 shows a comparison of baseline characteristics among study arms. Most of the patients were married females. The CHW and control study samples had similar demographic and risk factors, although cigarette smoking was more prevalent in the intervention arm. There was a mean reduction in adjusted HbA1c of 0.53 percentage points in the CHW group compared to the control group, from which we estimated a gain of 0.04505 QALYs.

**Table 1:** Comparison of baseline characteristics of 268 patients enrolled in DCAS, American Samoa, 2009–2010

Characteristic	CHW arm *n* = 104	Usual care arm *n* = 164	Total *n* = 268	*p* value
Age (years)	56 ± 12	54 ± 13	55 ± 13	0.39
Married	82 [79]	129 [79]	211 [79]	0.9
Females	60 [58]	108 [66]	168 [63]	0.18
Risk level				0.21
Risk level low	10 [9.6]	20 [12.2]	30 [11]	
Risk level moderate	50 [48.1]	61 [37.2]	111 [41]	
Risk level high	44 [42.3]	83 [50.6]	127 [47]	
Current smoker	15 [14]	11 [7]	26 [10]	0.03
Biological measures				
BMI (kg/m^2^)	36 ± 7.3	37 ± 7.9	37 ± 7.7	0.88
Systolic BP (mmHg)	131 ± 16	134 ± 17	132 ± 17	0.25
Diastolic BP (mmHg)	84 ± 9.3	84 ± 11	84 ± 10	0.92
HbA1c (%)	9.6 ± 2.1	10 ± 2.3	9.8 ± 2.3	0.15

Note: The Diabetes Care in American Samoa (DCAS) intervention was a cluster-randomized, culturally tailored, home-visiting CHW intervention. Results are shown as number (percentage) or mean ± standard deviation. *P* values are based on the chi-square test or Fisher exact test for categorical variables and *t* tests for continuous. Risk level is based on HbA1c, BP, smoking status, alcohol consumption, and depression (PHQ-9 score)

*CHW* community health worker, *BMI* body mass index [weight (kg)/height (m^2^)], *BP* blood pressure (mmHg), *HbA1c* glycated hemoglobin (%)

Table 2 shows the base case scenario where we found the intervention had a net cost of $594.27 per patient. Our ICER for our primary outcome—HbA1c reduction—is $1121.26 per percentage point reduction in HbA1c. Our ICER using QALYs is $13 191.24 per QALY gained.

**Table 2:** Incremental cost-effectiveness

	Costs	Effectiveness		Cost-effectiveness	
	Incremental costs	HbA1c reduced	QALYs gained	ICER (HbA1c)	ICER (QALYs)
Intervention	$594.27	0.53	0.05	$1121.26	$13 191.24

Note: Incremental costs shown in Table 3

We estimated direct intervention costs to be $677.43 per patient: $439.18 for staffing costs, $124.24 for equipment usage, and $52.94 for donated space from TFHC.

Table 3 shows that, over the 12-month follow-up period, hospitalization costs in the intervention arm increased by $19.29 per patient. Overall, those in the intervention arm stayed 0.14 fewer inpatient days per patient. Despite this greater decrease compared to the control group, a slight increase in the intervention group of 0.06 inpatient days in the ICU—a more expensive unit—outweighed the cost reduction in other hospital wards. ED costs in the intervention arm were reduced by $83.77 per patient. Outpatient costs were reduced by $122.15 per patient. Per patient visit, ED costs in the base case were actually lower than per patient visit costs in any primary care ambulatory clinic.

**Table 3:** Two-year service utilization and cost difference in differences from baseline relative to control arm

	Utilization DiD	Cost DiD
Intervention direct		
Intervention (use)	1.00	$677.43
Medical direct		
ED (visits)	− 0.61	− $83.77
Ambulatory (visits)	− 0.18	− $38.38
Hospitalizations (inpatient days)	− 0.14	$19.29
Surgery	− 0.10	− $12.92
Indirect patient time		
Intervention indirect (hours)	10.03	$47.76
Medical indirect (hours)	− 3.74	− $15.15
Total		$594.27

Note: Utilization difference in differences (DiD): mean differences in pre-post service use of community health worker (CHW) arm to control arm. Positive numbers are service increases in the CHW arm. Total differences are divided by follow-up person-years (p-y) in each intervention arm [CHW 100 p-y, control 163 p-y [14]]. Cost difference in differences: the difference in cost per patient per year, derived by multiplying utilization differences by the cost of each utilization output (not displayed). DiD in costs from the baseline period (− 12 to 0 months) for each patient relative to control across the intervention time period (0 to 12 months). All costs in 2012 USD ($). See Additional file 1: Table S1 for further medical cost breakdown. Intervention direct: mean difference is participation in the intervention: since all intervention arm patients received (CHW) home visits, the mean difference is 1. Cost is per participation in the program based on staffing, equipment usage, donated space, and consumables. Medical direct: four broad categories. Ambulatory visits are summed across internal medicine clinic, primary care clinic, community health center, ophthalmology, surgical clinic, mental health clinic, and ENT. Hospitalizations are summed across inpatient days in the medical ward, surgical ward, and ICU. Please see Additional file 1: Table S1 for further breakdown. Indirect patient time: “intervention indirect” denotes average time spent by the patient during home visits. “Medical indirect” includes time spent during ED and ambulatory visits (estimated at 1/2 h) and during hospital inpatient days (estimated at 24 h). Costs calculated at the minimum wage of $4.76 per hour plus time-and-a-half if over 8 h (Department of Labor). Total: net total difference in costs per patient per year summed across all cost differences. This is the incremental cost difference

Patients in the intervention arm spent a mean of 602 min on intervention activities. The indirect patient costs of participating in the intervention were $47.76 per patient for the year. Indirect patient costs from changes in healthcare utilization were $15.15 less per patient in the intervention arm, largely because reduced LOS took up less patient time.

Sensitivity analyses are shown in Additional file 1: Table S2. Our ICER results are sensitive to one-way changes in the relationship between HbA1c and QALY weights. Using the most extreme values, the ICER increased up to $74 750.36/QALY, greater than the most conservative threshold of 3× GDP per capita in the local setting of $39 000/QALY gained and the more commonly used threshold of $50 000/QALY gained; still below, however, other willingness-to-pay thresholds of 2× and 3× GDP per capita of $102 902 and $154 353/QALY gained.

## Discussion

We found the DCAS intervention was highly cost-effective, as our ICER of about $13k/QALY gained was considerably below any acceptable willingness-to-pay threshold. We focus the discussion on QALY-based measures since ICERs using disease-specific outcomes—such as HbA1c reduction—can only be compared to other T2DM interventions and cannot provide theoretically sound thresholds for determining cost-effectiveness. ICERs using QALYs can be compared across a wide range of interventions and diseases and can be used with theoretically sound thresholds for determining cost-effectiveness [17]. Our base case ICER of $13 191.24 per QALY gained is well below the commonly used threshold of $50 000 /QALY for cost-effectiveness and far below other thresholds including 2× and 3× GDP per capita/QALY [36], as well as below other study-derived willingness to pay thresholds of $66 351 and $111 134/QALY [38, 39]. This ICER is within the range found in a similar study [11], although our study may offer an improved approach since the RCT used a control group as a direct comparator.

Less expensive health care labor in American Samoa, including that of doctors, limits our cost-effectiveness analysis to low- and middle-income country (LMIC) settings when using a willingness-to-pay threshold of $50 000. However, even with a far more conservative setting-specific threshold of 3xGDP per capita/QALY gained (which would account for less expensive labor costs), the intervention is still highly cost-effective since GDP per capita in Samoa is $13 000 [31].

Our modeled cost-utility results are conservative for at least three important reasons. First, our sample has a higher baseline HbA1c level than the sample we used to model QALYs gained from HbA1c reductions [40]. QALYs might not increase linearly with HbA1c reductions. Reductions in higher baseline HbA1c levels seem to lead to greater improvements in health outcomes, and thus greater QALYs gained for the same percentage point reduction in HbA1c [42, 43]. A similar study found their intervention to be most cost-effective for participants with the highest baseline HbA1c [11]. Since we modeled QALYs to increase linearly with the decrease in HbA1c, decreases in HbA1c at higher baseline levels of HbA1c could potentially yield greater QALY gains. Greater QALY gains than those modeled here would further reduce our ICER. Second, longer time horizons may also reduce our ICER as patients in each arm have more time to experience adverse events [28]. In the intervention with low-income Mexican-Americans with T2D, study authors found that the longer the time horizon, the lower the QALY-denominated ICER, with the ICER at the 5-year horizon larger than the ICER at the 20-year horizon by almost a factor of 4 [11]. Third, we believe our final cost-effective result to be robust because we were conservative in our selection of base case costs. We selected the most costly option to estimate donated rent of TFHC space for the intervention, and we valued outpatient clinic visits at the community health center at the same price as the hospital outpatient clinic, although we expect health center costs to be lower. Our less conservative selections included using peak intervention output for overhead costs, which is reasonable for modeling how the intervention would operate if it were an ongoing program.

We can also compare our HbA1c-denominated ICER with studies also estimating such ICERs. Our ICER is higher than the ICER found in a CHW intervention in a Black American population by a factor of more than 7, which might imply our intervention model is less cost-effective [18]. That study, however, did not consider the costs of healthcare utilization, continued physician involvement with those in the intervention arm, or the opportunity costs of space and equipment.

Our finding that ED care is cheaper per patient than regular outpatient clinic care runs counter to general perceptions. Quality of care and care linkage might be worse in an ED, and ED charges are often quite high for consumers. However, when examined from a resource utilization perspective—instead of hospital charges—it is unclear why ED visits are more expensive than regular visits. Although doctors in our study self-reported spending about double the amount of time with a patient in the ED than in an outpatient setting, we find that nurses, staff, other medical resources, and space were more efficiently used in the ED than in outpatient settings. Observations of the community health center suggest that the clinic was understaffed during the usual and diabetes-specific primary care hours [14].

This study is limited by our modeling methodology for the impact of QALYs per one percentage point HbA1c reduction, which was modeled on a different LMIC population in a cross-sectional study. Since T2DM is already quite high in American Samoa, there was likely already established pathology and some end-organ disease in our participants so that some medical care costs for patients may have been beyond the ability of our CHW intervention to attenuate. Since HbA1c is a surrogate and not an ultimate clinical endpoint, induced changes in HbA1c might not elicit the same quality of life changes as expected from a cross-sectional design. Interventions can also impact the quality of life beyond clinical factors. Post-intervention focus groups with participants indicated broadly positive experiences about being in the study and the care provided by the CHWs, and many tried to continue seeing the CHWs even after the study concluded. Future studies of CHW interventions might focus on the quality of life and well-being to complement the biomedical and cost-effectiveness measures.

## Conclusions

Sustaining health interventions—even those shown to be cost-effective and culturally relevant—is particularly challenging in under-resourced and low-income environments [44]. In our DCAS study, HbA1c levels in the intervention group stabilized 2 years after study completion, but those in the control group returned almost to baseline in the year following receipt of the intervention [45]. While there is no single remedy to promote subsequent investment in health programs, scholars who are committed to improving public health should work closely with local policymakers to encourage continued investment in proven interventions, acknowledging the very real financial and human resource constraints in these settings.

Compared to multiple willingness-to-pay thresholds ranging from $39 000 to $111 134 per QALY gained, this ICER shows that the CHW intervention is highly cost-effective. Future studies of the cost-effectiveness of CHW T2DM interventions in similar settings should model lifetime costs and QALYs gained to more accurately assess long-term cost-effectiveness. Data on health-related quality of life (HR-QOL), indirect costs, and utility preferences for different HR-QOL health states varying by societal context should also be collected. Studies should also strive for concurrent collection of medical utilization patterns and direct medical costs, as these are essential for robust cost-effectiveness analysis.

The burden of T2DM-related disease in American Samoa is unacceptably high. Given the success of the DCAS intervention in providing culturally competent, highly cost-effective care to improve T2DM self-management, external and local funders of the healthcare system should re-invest in the CHW program to help improve the health of this underserved population.

## Additional file

Additional file 1: **Table S1.** Direct medical costs. **Table S2.** Sensitivity analyses. (DOCX 18 kb)
