# Supplementary material for: Cost-effectiveness analysis of a cluster-randomized, culturally tailored, community health worker home-visiting diabetes intervention versus standard care in American Samoa
Source: Hum Resour Health. 2019 Mar 5;17:17. doi: 10.1186/s12960-019-0356-6 (PMC6402127; doi:10.1186/s12960-019-0356-6)
Supplement: Supplementary file 1 — Table S1. Direct medical costs. Table S2. Sensitivity analyses. (DOCX 18 kb) [file 12960_2019_356_MOESM1_ESM.docx]

Supplementary Table 1 – Direct Medical Costs

|  | CHW | Mean CHW | Control | Mean Control | Mean Diff. output | Cost per output | Cost  Diff. |
| --- | --- | --- | --- | --- | --- | --- | --- |
| ED | -54 | -0.54 | 11 | 0.07 | -0.61 | $137.90 | -$83.77 |
| Int Med Clinic | -18 | -0.18 | 2 | 0.01 | -0.19 | $148.51 | -$28.55 |
| TFHC | -2 | -0.02 | -13 | -0.08 | 0.06 | $148.51 | $8.87 |
| Primary Care Clinic | -7 | -0.07 | 6 | 0.04 | -0.11 | $215.53 | -$23.02 |
| Ophthalmology | 18 | 0.18 | 47 | 0.29 | -0.11 | $186.89 | -$20.25 |
| Surgical Clinic | 20 | 0.20 | -5 | -0.03 | 0.23 | $256.43 | $59.15 |
| Psych clinic | -8 | -0.08 | 2 | 0.01 | -0.09 | $435.29 | -$40.16 |
| Eye emergency referrals | 0 | 0.00 | 2 | 0.01 | -0.01 | $186.89 | -$2.29 |
| ENT | -1 | -0.01 | -9 | -0.06 | 0.05 | $174.19 | $7.88 |
| LOS ACUTE | 5 | 0.05 | 44 | 0.27 | -0.22 | $565.87 | -$124.46 |
| LOS SURGICAL | 0 | 0.00 | -4 | -0.02 | 0.02 | $487.70 | $11.97 |
| LOS ICU | -3 | -0.03 | -14 | -0.09 | 0.06 | $2,357.89 | $131.78 |

Note. ED=emergency department; TFHC= Tafuna Family Health Centers; ENT=ear, nose and throat; LOS=length of stay; ICU=intensive care unit

Supplementary Table 2 – Sensitivity Analyses

| Factor | Lowest  ICER | Highest  ICER |
| --- | --- | --- |
| 1-way HbA1c | $7,424.98 | $59,048.63 |
| 1-way QALY weight | $11,212.55 | $74,750.36 |
